# High colored dissolved organic matter (CDOM) absorption in surface waters of the central-eastern Arctic Ocean: Implications for biogeochemistry and ocean color algorithms

**DOI:** 10.1371/journal.pone.0190838

**Published:** 2018-01-05

**Authors:** Rafael Gonçalves-Araujo, Benjamin Rabe, Ilka Peeken, Astrid Bracher

**Affiliations:** 1 Phytooptics Group, Physical Oceanography of Polar Seas, Climate Sciences Division, Alfred Wegener Institute Helmholtz Centre for Polar and Marine Research, Bremerhaven, Germany; 2 Faculty of Biology and Chemistry (FB-2), University of Bremen, Bremen, Germany; 3 Physical Oceanography of Polar Seas, Climate Sciences Division, Alfred Wegener Institute Helmholtz Centre for Polar and Marine Research, Bremerhaven, Germany; 4 Polar Biological Oceanography, Biosciences Division, Alfred Wegener Institute Helmholtz Centre for Polar and Marine Research, Am Handelshafen 12, Bremerhaven, Germany; 5 Institute of Environmental Physics, University of Bremen, Bremen, Germany; CNRS, FRANCE

## Abstract

As consequences of global warming sea-ice shrinking, permafrost thawing and changes in fresh water and terrestrial material export have already been reported in the Arctic environment. These processes impact light penetration and primary production. To reach a better understanding of the current status and to provide accurate forecasts Arctic biogeochemical and physical parameters need to be extensively monitored. In this sense, bio-optical properties are useful to be measured due to the applicability of optical instrumentation to autonomous platforms, including satellites. This study characterizes the non-water absorbers and their coupling to hydrographic conditions in the poorly sampled surface waters of the central and eastern Arctic Ocean. Over the entire sampled area colored dissolved organic matter (CDOM) dominates the light absorption in surface waters. The distribution of CDOM, phytoplankton and non-algal particles absorption reproduces the hydrographic variability in this region of the Arctic Ocean which suggests a subdivision into five major bio-optical provinces: Laptev Sea Shelf, Laptev Sea, Central Arctic/Transpolar Drift, Beaufort Gyre and Eurasian/Nansen Basin. Evaluating ocean color algorithms commonly applied in the Arctic Ocean shows that global and regionally tuned empirical algorithms provide poor chlorophyll-*a* (Chl-*a*) estimates. The semi-analytical algorithms Generalized Inherent Optical Property model (GIOP) and Garver-Siegel-Maritorena (GSM), on the other hand, provide robust estimates of Chl-*a* and absorption of colored matter. Applying GSM with modifications proposed for the western Arctic Ocean produced reliable information on the absorption by colored matter, and specifically by CDOM. These findings highlight that only semi-analytical ocean color algorithms are able to identify with low uncertainty the distribution of the different optical water constituents in these high CDOM absorbing waters. In addition, a clustering of the Arctic Ocean into bio-optical provinces will help to develop and then select province-specific ocean color algorithms.

## 1. Introduction

The Arctic Ocean basin receives 11% of the global freshwater input with its volume representing only 1% of the global ocean [1]. It obtains the largest amount of freshwater relative to its volume and therefore is the ocean most influenced by the continents on Earth. Together with the fresh water, high loads of terrestrial material (organic and inorganic; dissolved, colloidal and particulate) are introduced in that basin, in particular through the wide Siberian continental shelves [2–6]. By this the Arctic Ocean presents a large carbon reservoir and plays an important role in the planet’s carbon cycle. Besides, the Arctic environment has been experiencing the effects of ongoing global warming regarding permafrost thaw [7], changes in fresh water export [8,9] and decline of sea-ice extent [10,11] and volume [12]. The permanent loss of sea-ice may lead to an increase in light penetration in the Arctic surface layer [13] and to changes in the composition of phytoplankton assemblages [14], the overall primary production in the Arctic Ocean [15,16], and the degradation of terrestrial material transported to that basin [17,18].

Recent studies have pointed out regional differences in the Arctic Ocean with respect to biogeochemical parameters. For instance, shelf and open Arctic seas have shown to diverge in regards to the fluxes of biogenic matter [4] and export of terrigenous material [3]. Furthermore, while varying between shelf and open water in the Arctic [3,19], colored and fluorescent dissolved organic matter (CDOM and FDOM, respectively) content also differ between the western and eastern Arctic seas [6,20–23]. Similarly, geographical differences in primary [24,25] and net community production [26], as well as in phytoplankton (e.g., dinoflagellates) and protist distribution [27,28] in the various basins of the Central Arctic Ocean have been observed. Such biogeographic patterns are likely related to hydrographic and sea-ice conditions within the region [28,29], denoting a strong coupling of physical and biogeochemical processes within the surface layers of the Arctic Ocean.

With the aforementioned effects of global warming and its impacts on the Arctic environment, improved monitoring and understanding of the current situation and changes in biogeochemical parameters are necessary. The optical properties of dissolved organic matter are reliable water mass tracers in the Arctic Ocean according to reports based on *in situ* [22,30] and remote sensing data [31] and has also been proven to be useful on monitoring small scale changes in coastal environments [32]. Biogeochemical parameters such as chlorophyll-*a* (Chl-*a*) and CDOM can be determined (and be estimated for primary production) in surface waters by ocean color remote sensing. Furthermore, the Arctic Ocean is a unique ocean where, even in pelagic waters, the non-water light absorption in the surface layer being dominated by CDOM [21] which does not co-vary with Chl-*a*. Opposed to that, the latter is assumed by empirical ocean color algorithms. Hence, these algorithms lead to an overestimation of Chl-*a* [33–35] and overall poor performance in the Arctic [36]. Improvement of algorithms for the Arctic Ocean is challenging given the difficulties to sample for validation data in those waters, in particular, on the remote Siberian shelves [37]. Several studies have addressed the quality of the estimates from ocean color algorithms in the western Arctic ocean [19,21,33–35,38,39]. Regionally tuned algorithms provided improved estimates related to global algorithms in the western Arctic [34,38]. In that same region, semi-analytical algorithms obtained even better estimates of Chl-*a* [21,33]. Besides Chl-*a*, semi-analytical algorithms can also retrieve CDOM in that region with low uncertainty [19,21,33].

Here, we focus on optical and hydrographic sampling in the central-Eastern Arctic, an area up to now hardly evaluated [39] for the application of satellite and *in situ* optical measurements to monitor the surface biogeochemistry of the Arctic Ocean. The objectives of this study are twofold: first, we aim to obtain a characterization of the non-water absorption constituents in the surface waters in the Central-Eastern Arctic. Those properties were tested whether they reproduce hydrographic and geographic patterns (or units). As a second objective, we evaluate empirical and semi-analytical ocean color algorithms commonly applied to studies in the Arctic Ocean and compare their performances. Given the novelty of the results presented in this study, it contributes to the growing Arctic remote sensing research, which has been so far mostly devoted to the western Arctic Ocean. Moreover, as already pointed out [37], the sampling effort for the Arctic Ocean is still very low compared to other ocean basins and more studies are required to improve the ocean color estimates for that basin. Finally, it is important to stress that whilst ocean color sensors are not able to monitor under very low (or no) illumination and cloudy conditions and ice-covered regions, *in situ* bio-optical measurements in those regions are crucial for improving biogeochemical models; however, such measurements are very scarce in the central and eastern Arctic Ocean. Furthermore, results on *in situ* bio-optical and biogeochemical properties are important for calibrating sensors coupled to autonomous platforms (e.g. satellites; gliders; Autonomous Underwater Vehicles, AUVs; Ice-Tethered Platforms, ITPs; etc.). In the future, those sensors will measure *in situ* biogeochemical properties enabling monitoring on high spatial and temporal resolution and coverage in the Arctic Ocean [e.g., ITPs [40–42]].

## 2. Methods

### 2.1. Sampling

**Fig 1:**
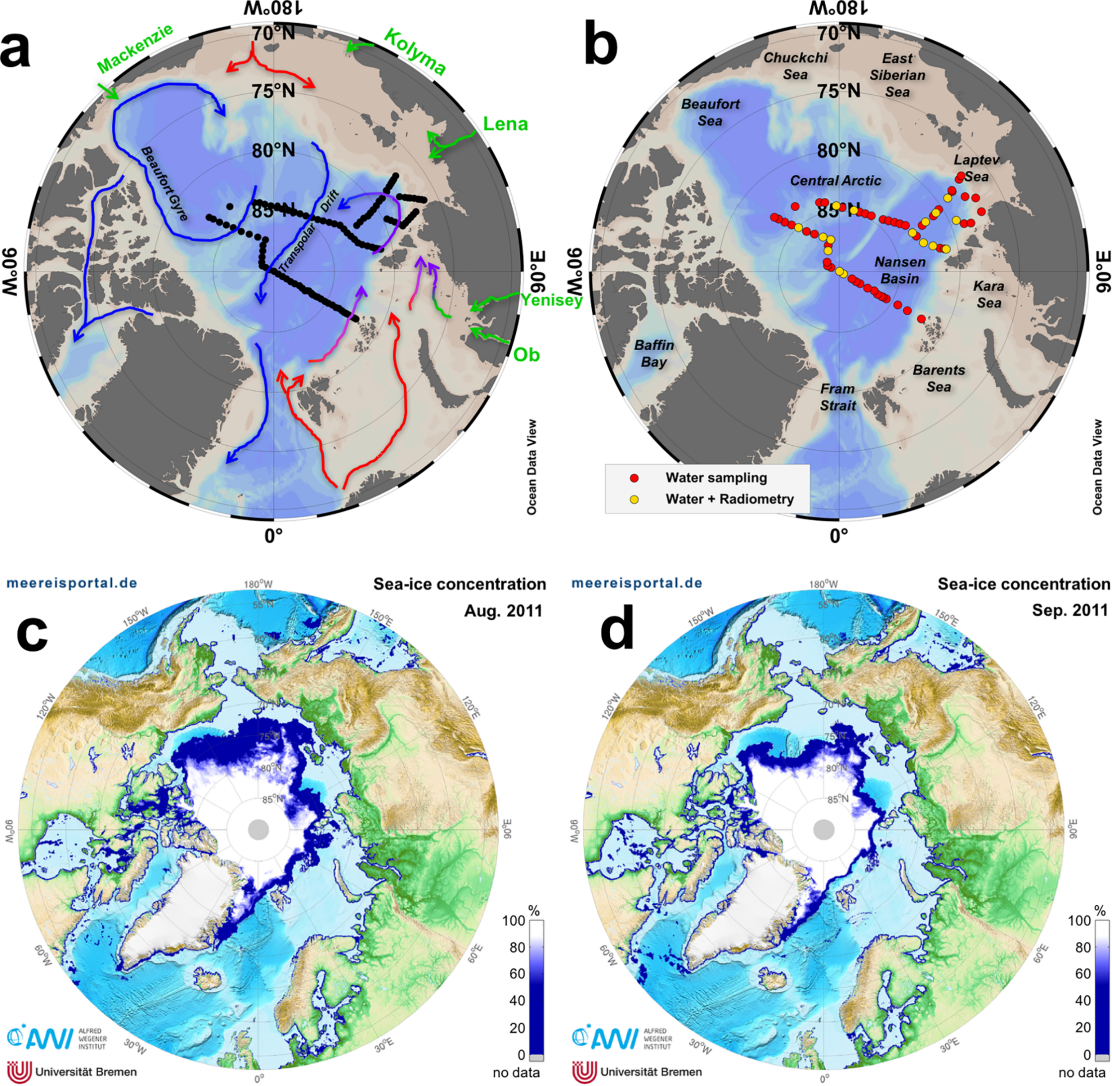
Study region and sea-ice conditions. ODV maps [44] showing the sampling stations occupied during the ARK-XXVI/3 (PS-78) cruise where CTD casts **(a),** water sampling and hyperspectral radiometric measurements **(b)** were performed. Arrows in (a) represent the main surface circulation patterns in the Arctic Ocean colored as follows: major rivers (green); inflowing currents (red); out flowing currents (blue) [45]. AMRSR-2 sea-ice concentration (http://meereisportal.de) for August **(c)** and September **(d)** 2011.

The ARK XXVI-3 (PS-78) cruise was conducted in shelf and open waters through the central-eastern Arctic Ocean from 5^th^ August to 6^th^ October 2011 onboard the *R*/*V* Polarstern. Temperature and salinity profiles were acquired with a CTD attached to a rosette system at 110 oceanographic stations [43] (Fig 1A). Surface water samples for analysis of dissolved organic matter, particulate matter and chlorophyll-*a* (Chl-*a*) were taken using Niskin bottles attached to the rosette system at 62 stations (Fig 1B). As observed in Fig 1C and 1D, most of the sampled area was covered by sea-ice. No specific permissions were required for these locations/activities given that sampling was performed out of the 200 Mile zone. Data are available at https://doi.org/10.1594/PANGAEA.867532.

### 2.2. Particulate absorption analysis

Water samples for particulate absorption analysis were filtered on GF/F filters (0.7 μm pore size), shock-frozen in liquid nitrogen and stored at –80°C until laboratory analysis at the Alfred Wegener Institute Helmholtz Centre for Polar and Marine Research. Measurements were carried out on a dual-beam UV/VIS spectrophotometer (Cary 4000, Varian Inc.) equipped with a 150 mm integrating sphere (external DRA-900, Varian, Inc. and Labsphere Inc., made from Spectralon^TM^) using a quantitative filterpad technique [46]. The filters were placed inside and at the center of the integrating sphere using a center-mount filter holder perpendicular to the light beam. A wavelength scan from 300 to 850 nm with a resolution of 1 nm (slit width 2 nm, scan rate 150 nm min^**−**1^) was performed, when the reflectance ports were covered with Spectralon^TM^ reflectance standards. The baseline was recorded beforehand with a clean, dry filter, and a filter, which was soaked for more than 30 min in freshly produced Milli-Q water, was taken as a reference. The absorption coefficient was calculated from the transmittance [*T*(λ)], which is derived from the optical density (OD) measurements, using a path length amplification factor of 4.5 (*β* = 1/4.5) [47] following the equation:

$$a_{p}(\lambda )[m^{-1}]=-ln[T(\lambda )\times A\times \beta \times V^{-1}],$$ (1)

T(λ)=exp[−OD(λ)], (2)

where *V* is the filtrated sample volume in m^3^ and *A* the filter clearance area in m^2^. Results from the original filter gave total particulate absorption, *a*_p_. The algal pigments were bleached with NaOCl [48,49] to determine the absorption by detrital material (or non-algal particles), hereafter referred to as non-algal particles (*a*_NAP_). The bleached filters were measured following the procedure described above. The particulate absorption of phytoplankton at each wavelength (λ) [*a*_ph_**(**λ**)**] was obtained by subtracting *a*_NAP_ from *a*_p_.

### 2.3. Dissolved organic matter absorption analysis

Water samples for DOM analysis were filtered through prerinsed 0.2 μm filters immediately after sampling and stored in amber glass vials in dark at 4°C until analysis in laboratory at the Alfred Wegener Institute Helmholtz Centre for Polar and Marine Research after the cruise. CDOM was analyzed with an Aqualog® fluorescence spectrometer (HORIBA Jobin Yvon, Germany) using freshly produced Milli-Q water as reference. CDOM absorbance spectra measurements (260–600 nm) were blank-corrected and a baseline correction was applied at 600 nm, assuming negligible CDOM absorption at that wavelength. CDOM absorbance was further converted into Napierian absorption coefficient [*a*_CDOM_(λ)], obtained from the given equation:

$$a_{CDOM}(\lambda )[m^{-1}]=\frac{\left[2.303\times A(\lambda )\right]}{L},$$ (3)

where *A*(λ) is the absorbance at specific wavelength and *L* is the cuvette path length in meters. *a* is generally adopted as a proxy for assessing the CDOM content in a given water sample and in this study it is presented in the visible [440 nm—*a*_CDOM_(440)] and UV [350 nm—*a*_CDOM_(350)] bands. *a*_CDOM_(440) was chosen given its application to ocean color remote sensing [50,51] and to make it comparable with the particulate matter absorption coefficients [52]. The UV band *a*_CDOM_(350) was determined in this study due to its correlations to DOC and lignin concentrations and to permit comparison with earlier results [6,20,53].

### 2.4. Chlorophyll-*a* analysis

For measuring the photosynthetic pigment Chl-*a* one liter of seawater samples were taken from Niskin bottles, immediately filtered on GF/F filters, frozen in liquid nitrogen, and stored at -80°C until further analyses by high-performance liquid chromatography (HPLC) at the home laboratory of the Alfred Wegener Institute Helmholtz Centre for Polar and Marine Research after the cruise. The samples were measured using a Waters 600 controller equipped with an auto sampler (717 plus), a photodiode array detector (2996) and the EMPOWER software. Chl-*a* was analyzed by reverse-phase HPLC using a VARIAN Microsorb-MV3 C8 column (4.6 3 100 mm) and HPLC-grade solvents (Merck). The solvents gradient and routine of analysis are fully described in Taylor et al. [54]. Chl-*a* concentrations were quantified based on peak area of the external standard, which was spectrophotometrically calibrated using extinction coefficients published in Jeffrey et al. [55].

### 2.5. Radiometric measurements

Underwater optical light fields were assessed through radiance and irradiance profiles obtained with hyperspectral radiometers (RAMSES, ARC-VIS and ACC-VIS, respectively, TriOS GmbH, Germany). The instruments cover a wavelength range of 320 nm to 950 nm with an optical resolution of 3.3 nm and a spectral accuracy of 0.3 nm. Measurements were collected with sensor-specific automatically adjusted integration times (between 4 ms and 8 s). 16 radiometric profiles (Fig 1B) were collected simultaneously with the CTD profiles down to a maximum depth of 100 m. At each profile, measurements of upwelling radiance (*L*_u_) and downwelling irradiance (*E*_d_) were performed. One of the in-water sensors was equipped with inclination and pressure sensors. To avoid ship shadow, the ship was oriented such that the sun was illuminating the side where the measurements have taken place.

The radiometric measurements were performed out of the ship’s shadow and during clear sky or nearly clear sky conditions; this was checked based on the ship’s global radiation sensor data ensuring low variation of the incoming sunlight. For the in-water data, the inclination in either dimensions was smaller than 14° [35]. During the acquisition of the profiles, stops (varying from 30 to 60 s) were performed within a 10 m depth interval. These data were then averaged in discrete intervals of 5 and 10 m for 0–30 m and below, respectively, and were further processed following the NASA protocols [56]. As surface waves strongly affect measurements in the upper few meters, deeper measurements that are more reliable to be used can be further extrapolated to the sea surface [56]. Analogously to Stramski et al. [57] a depth interval was defined (***z***’ = 10 to 30 m) to calculate the vertical attenuation coefficients for downwelling irradiance and upwelling radiance, [i.e. *K*_d_(λ,*z*’) and *K*_u_(λ,*z*’), respectively]. With *K*_d_(λ,*z*’) and *K*_u_(λ,z’), the subsurface irradiance *E*_d_^−^(λ, 0 m) and radiance *L*_u_^−^(λ,0 m) were extrapolated from the profiles of *E*_d_(λ,*z*) and *L*_u_(λ,*z*).

For the calculation of the remote sensing reflectance [*R*_rs_(λ)], the subsurface *L*_u_^−^(λ, 0 m) and *E*_d_^−^(λ, 0 m) were propagated through the water-air interface by applying a transfer coefficient of 0.519 [57]. *R*_rs_(λ) was then calculated:

$$R_{rs}(\lambda )=\frac{\left[0.519\times L_{u}^{-}(\lambda ,0\;m)\right]}{E_{d}^{-}(\lambda )}.$$ (4)

### 2.6. Ocean color algorithms

In this study we evaluated the performance of ocean color algorithms to derive Chl-*a*, *a*_CDOM_(λ), *a*_dg_(λ) [the sum of *a*_CDOM_(λ) and *a*_NAP_(λ)] and *a*_ph_(λ). Firstly, we tested different empirical algorithms, which are used to derive Chl-*a* from band ratios of *R*_rs_. These algorithms are frequently applied to the Arctic Ocean. Here their Chl-*a* retrievals were obtained using *R*_rs_ from the 16 stations as input and then compared with *in situ* measured Chl-*a*. The MODIS OC3M is a global algorithm, which is determined as a function of three *R*_rs_ band ratios [58]. The global SeaWiFS OC4V6 [58,59] and the regional Arctic OC4L [38] algorithms, nevertheless, use a four-band ratio approach. These algorithms are expressed as follows:

$$\begin{array}{l} \mathrm{Chl}\left(\mathrm{OC}3M\right)=10^{\left(a+bR’+cR{’}^{2}+dR{’}^{3}+eR{’}^{4}\right)}\\ \;R’=\log\left[R_{\mathrm{rs}}\left(443>488/551\right)\right]\\ \;a=0.2830,b=-2.753,c=1.457,d=0.659,e=-1.403, \end{array}$$ (5)

$$\begin{array}{l} \mathrm{Chl}\left(OC4V6\right)=10^{\left(a_{1}+b_{1}R+c_{1}R^{2}+d_{1}R^{3}+e_{1}R^{4}\right)}\\ \;R=\log\left[R_{\mathrm{rs}}\left(443>490>510/555\right)\right]\\ \;a_{1}=0.366,b_{1}=-3.067,c_{1}=1.930,d_{1}=0.649,e_{1}=-1.532, \end{array}$$ (6)

$$\begin{array}{l} \mathrm{Chl}\left(\mathrm{OC}4L\right)=10^{\left(a_{2}+b_{2}R\right)}\\ \;R=\log\left[R_{\mathrm{rs}}\left(443>490>510/555\right)\right]\\ \;a_{2}=0.592,b_{2}=-3.607, \end{array}$$ (7)

where *R* is the base 10 logarithm of the maximum band ratio, whichever is the greatest of *R*_rs_(443)/*R*_rs_(555), *R*_rs_(490)/*R*_rs_(555), and *R*_rs_(510)/*R*_rs_(555); *R*’ is the same as *R* but it considers the greater of the two band ratios *R*_rs_(443)/*R*_rs_(551) and *R*_rs_(488)/*R*_rs_(551); and the coefficients *a*, *b*, *c*, *d*, *e*, *a*_1_, *b*_1_, *c*_1_, *d*_1_, *e*_1_, *a*_2_, and *b*_2_ are empirically derived values. Additionally, the performance of modifications to the global OC3M and OC4V6 algorithms developed for the western Arctic Ocean [34] hereafter OC3M-mod and OC4V6-mod, respectively, was evaluated. The coefficients for those regional algorithms are given below:

- OC3M-mod: *a*_3_ = –0.32, *b*_3_ = –2.33, *c*_3_ = 4.02, *d*_3_ = –31.64, *e*_3_ = 48.54;
- OC4V6-mod: *a*_4_ = –0.35, *b*_4_ = –1.52, *c*_4_ = –2.44, *d*_4_ = –12.80, *e*_4_ = 30.48.

Apart from the empirical ocean color algorithms, two semi-analytical algorithms (SAA) were tested. First, we used the Generalized Inherent Optical Property model (GIOP) [60,61], for simplicity further named GIOP, using settings applied for the western Arctic [33] to allow comparison with the results from that study. In short, GIOP is a spectral matching inversion model, which applies non-linear least square methods to retrieve three eigenvalues [*a*_ph_(443), *a*_dg_(443) and the particles spectral backscattering coefficient–*b*_bp_(555)]. GIOP can also estimate Chl-*a* from *a*_ph_(443), by using the factor 0.055. As in Chaves et al. [33], we used the GIOP applied to *in situ R*_rs_(λ) at the SeaWiFS/MODIS-Aqua operational wavelengths (412, 443, 490, 510, 555 and 670 nm). Besides the GIOP, a modification of the Garver-Siegel-Maritorena (GSM) SAA [62,63] for retrieving *a*_CDOM_(λ) in the Arctic Ocean [19] was used. This algorithm was developed based on a parametrization of absorption properties using data from the western Arctic. In short, it enables the separation of *a*_NAP_(λ), and therefore *a*_CDOM_(λ), from *a*_dg_(λ) by applying the parametrization of *a*_NAP_(λ) related to the particle backscatter at 555 nm [*b*_bp_(555)] [35].

To summarize, in this study we evaluate the following retrievals from ocean color algorithms:

- Chl-*a*_OC3M_ [58];
- Chl-*a*_OC4V6_ [58,59];
- Chl-*a*_OC3M-mod_ and Chl-*a*_OC4V6-mod_ [34];
- Chl-*a*_OC4L_ [38];
- *a*_ph_(λ)_GIOP_, *a*_dg_(λ)_GIOP_, and Chl-*a*_GIOP_ [60,61];
- *a*_dg_(λ)_Mat_ and *a*_CDOM_(λ)_Mat_ [19].

### 2.7. Statistical analysis

Hierarchical cluster analysis using simple average linkage and Euclidean distance method was applied to classify both, a matrix containing hydrographic and inherent optical properties (IOPs) bulk properties (hereafter environmental matrix) and a matrix consisting of hyperspectral apparent optical properties (AOPs), into hydrographically (and geographically) coherent groups [64]. The purpose of applying such an approach was to test whether the AOPs, or a combination of hydrography and IOPs, are capable to trace the origin of waters masses as previously suggested in other regions of the Arctic Ocean [22,31]. The first matrix, named environmental matrix, consisted of surface measurements of temperature, salinity, *a*_CDOM_(443), *a*_NAP_(443) and *a*_ph_(443), which were normalized prior to analysis, by subtracting the mean value and then dividing by the standard deviation. The environmental matrix consisted of parameters, which in future can be derived from sensors mounted together on autonomous platforms. The hyperspectral AOP matrix consisted of the second derivative of *R*_rs_(λ)/*R*_rs_(555) that computes the changes in curvature of a given spectrum over a sampling interval or band separation [65]. For that, *R*_rs_ spectra were interpolated to the optimal range for band separation (435–510 nm), and a smoothing filter window of 27 nm was used for the derivative calculations [65]. Given that bio-optical parameters are generally log-normally distributed in natural environments [66] and also in this study, power functions were applied to evaluate the correlation between pairs of bio-optical parameters [67,68].

Kruskal-Wallis H tests were applied to compare variables between pairs of clusters, after being normality-tested with the Kolmogorov-Smirnov test. To evaluate the performance of the ocean color algorithms, *r*^2^, slope, intercept, root mean square error (RMSE), mean absolute error (MAE) and bias for each pair of variables were determined. The RMSE, MAE and bias were calculated as follows:

$$RMSE=\sqrt{\frac{\sum_{n=1}^{N}{\left[{log}_{10}Y-{log}_{10}X\right]}^{2}}{N}},$$ (8)

$$MAE=\frac{\sum_{n=1}^{N}\left|Y_{n}-X_{n}\right|}{N},$$ (9)

$$BIAS=\frac{\sum_{n=1}^{N}Y_{n}-X_{n}}{N},$$ (10)

where *Y* is the retrieved parameter (e.g. Chl-*a*, *a*_dg_, *a*_ph_) and *X* is the correspondent *in situ* measured parameter.

## 3. Results and discussion

### 3.1. Hydrography

**Fig 2:**
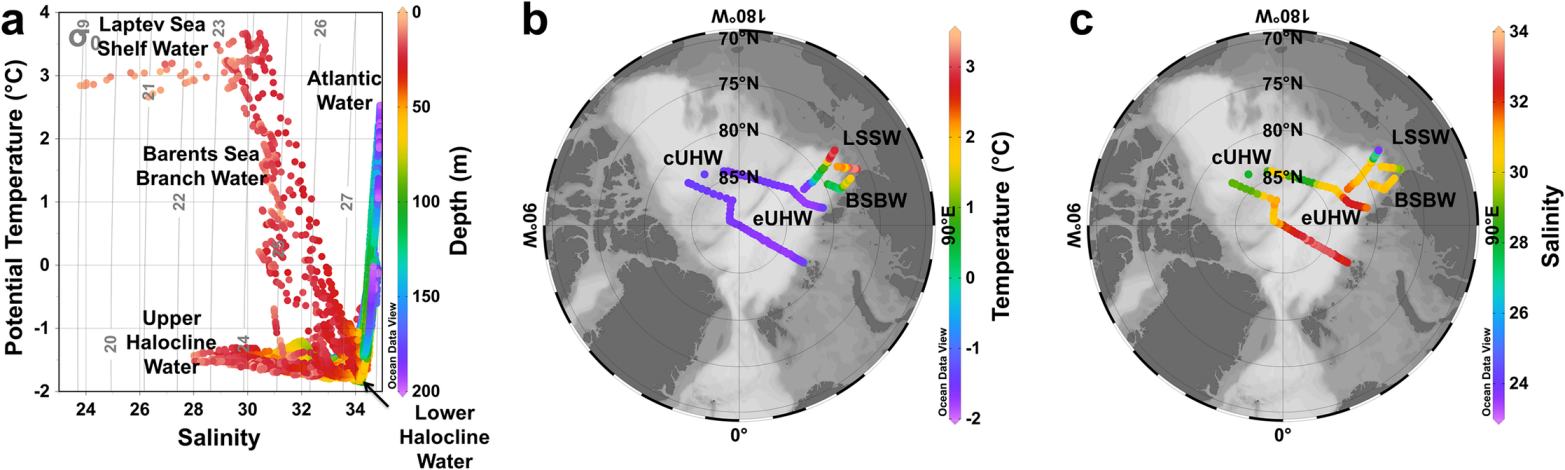
Hydrography in the surface central and eastern Arctic Ocean. **(a)** T-S diagram with depth (m) as color bar. Surface distribution of temperature (°C) **(b)** and salinity (**c**) with the approximate occupation of the water masses with the PML within the study region. Produced with Ocean Data View [44].

Based on the temperature and salinity profiles five water masses were identified within the surface layer (0−200 m) of the sampled area, which are in agreement with previous studies in the region [45,69]: Upper Halocline Water (UHW), Barents Sea Branch Water (BSBW) and Laptev Sea Shelf Water (LSSW) at the surface; and Lower Halocline Water (LHW) and Atlantic Water (AW) in the beneath layer (Fig 2A). Surface waters of the central Arctic were occupied by the UHW, whereas BSBW and LSSW were observed at surface in pelagic and shelf waters in the Laptev Sea, respectively. Most of the Arctic surface waters are of Atlantic origin and are progressively modified at higher latitudes by heat exchange with the atmosphere, river runoff, melt water in summer and salt rejection in winter [70]. The UHW was observed within the upper polar mixed layer (PML, ~40 m) and was characterized by subzero temperatures and a thin upper mixed layer (10–20 m) due to dilution from sea-ice melt. This water mass can be further divided into two origins, given differences in the salinity ranges [45]: the UHW from the Canadian Basin with the Beaufort Gyre (cUHW, with salinity <32.5), and the UHW from the Eurasian Basin (eUHW, with salinity between 32 and 34). The BSBW observed in the surface waters of the Laptev Sea is transported from the Kara Sea through the Vilkitsky Strait by the Vilkitsky Strait Current [69,71,72]. High temperatures and low salinity characterize the LSSW which is strongly influenced by the Lena River outflow [73]. The highest temperature (3.67°C) and lowest salinity (23.8) values were observed within LSSW. The lowest temperature (−1.86°C), on the other hand, was associated with LHW whereas the highest salinity (34.9) was observed within AW located in the deepest sampled layer, generally below 100 m (Fig 2B and 2C).

### 3.2. Absorption coefficients of water constituents

Phytoplankton absorption coefficients [*a*_ph_(443)] were highly correlated with the absorption coefficients of NAP [*a*_NAP_(443)] (*r*^2^ = 0.95; *p*<0.0001; *n* = 62). *a*_ph_(443) ranged from 0.01 to 0.06 m^-1^ whereas the *a*_NAP_(443) varied between 0.0004 and 0.04 m^-1^. The highest *a*_NAP_(443) values were associated with sites close to the shelf break, denoting the continent as its main source, reaching its maximum within the LSSW, in similar ranges as previously reported [21]. Relatively high values of *a*_ph_(443) were observed close to the Laptev Sea shelf break, as for *a*_NAP_(443), however, the highest *a*_ph_(443) values were obtained for the Nansen Basin. Further discussion on the spatial variability of those parameters is presented in Section 3.3.

**Fig 3:**
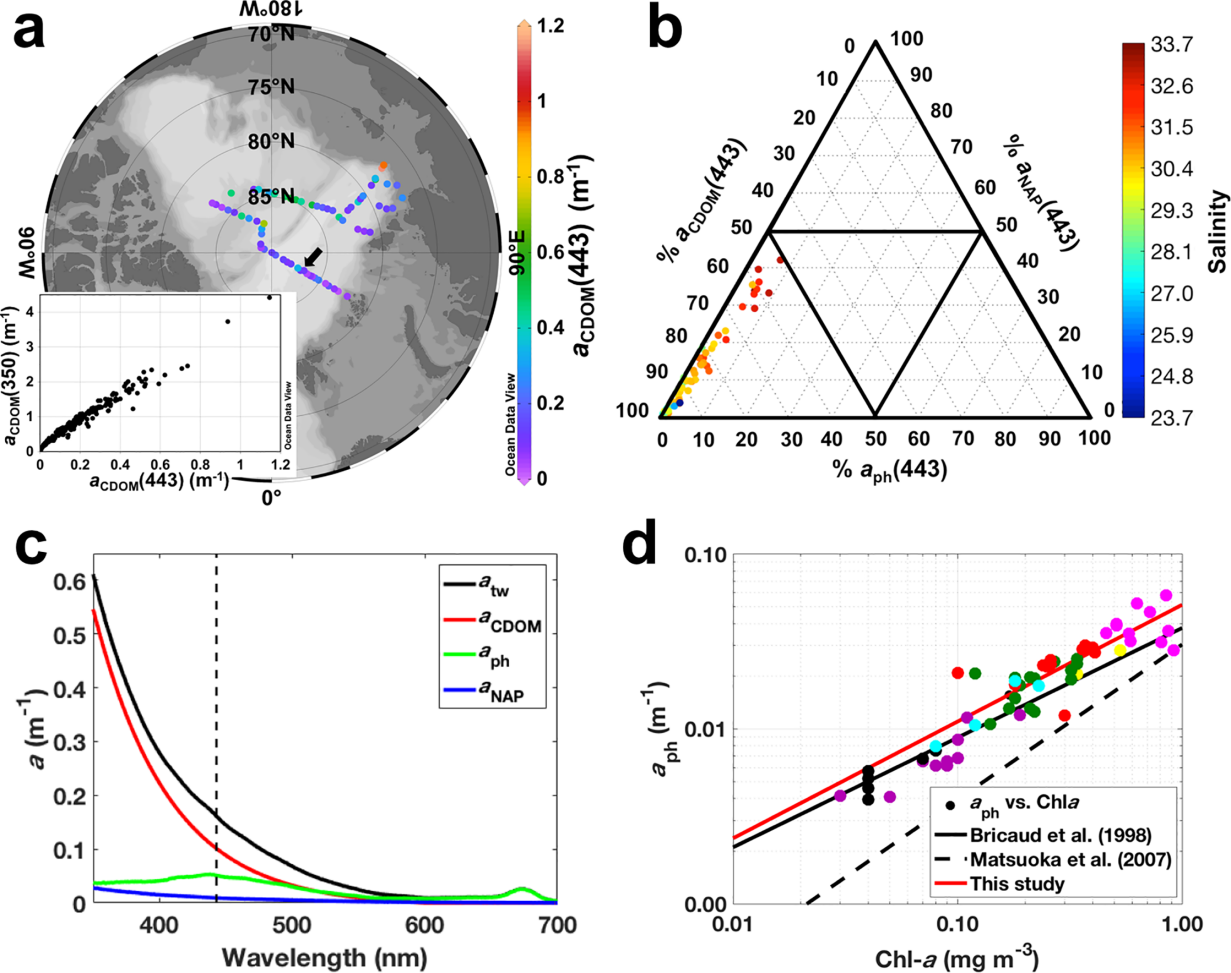
Absorption coefficients in the surface waters of central and eastern Arctic Ocean. **(a)** Surface distribution of *a*_CDOM_(443) (m^-1^) and correlation between *a*_CDOM_(443) and *a*_CDOM_(350) (inset graph); produced with Ocean Data View [44]. **(b)** Ternary plot denoting the contribution of the non-water absorbers [*a*_CDOM_(443), *a*_ph_(443), *a*_NAP_(443)] to total non-water absorption [*a*_tw_(443)] at surface; color bar indicates salinity. **(c)** Station 207 (indicated by the arrow in ***a***) as example of *a*_tw_(λ), *a*_CDOM_(λ), *a*_ph_(λ) and *a*_NAP_(λ) spectra (m^-1^). Dashed line indicates the position of 443 nm. **(d)** Correlation between Chl-*a* (mg m^-3^) and *a*_ph_(443) (m^-1^); for the colors, please refer to Fig 4.

To investigate the correlation of *a*_ph_(443) and *a*_p_(443) with Chl-*a* a power function was applied [67,68]. Both *a*_ph_(443) (Fig 3D) and *a*_p_(443) were highly correlated to Chl-*a*, however, as expected, the correlations for *a*_ph_(443) were higher. The power functions for *a*_ph_(443) and *a*_p_(443) in relation to Chl-*a* concentration obtained in this study are given below:

$$a_{\mathrm{ph}}(443)=0.0513{\left[\mathrm{Chl}-a\right]}^{0.6675}\;(r^{2}=0.85),$$ (11)

$$a_{p}(443)=0.0595{\left[\mathrm{Chl}-a\right]}^{0.5603}\;(r^{2}=0.73).$$ (12)

As shown in Fig 3D, the correlation between *a*_ph_(443) and Chl-*a* was comparable to the one found by Bricaud et al. [68], and to other expeditions conducted in the Fram Strait and adjoining seas [74–76]. The consistency between these results thus, reiterates the applicability of such absorption measurements in the VIS-range as a proxy to retrieve Chl-*a* concentrations. The correlation between *a*_ph_(443) and Chl-*a* observed in this study presents, in turn, a slight deviation from the trends reported for the Canadian Basin [35]. Since the authors claim that their deviation from the global average is caused by a different pigment packaging effect and/or pigment composition, in their specific region: this would in turn mean that for our data set the phytoplankton composition and light adaptations follow more the global average.

CDOM absorption coefficients in the visible and UV wavelength ranges [*a*_CDOM_(443) and *a*_CDOM_(350), respectively] were highly correlated (*r*^2^ = 0.99, *p*<0.0001) and ranged from 0.02 and 0.19 m^-1^ to 1.14 and 4.42 m^-1^, respectively (Fig 3A). The highest *a*_CDOM_(443) values [*a*_CDOM_(443)>1 m^-1^] were observed in the Laptev Sea associated to the LSSW, with values in similar ranges as previously reported for those waters [21,50,53]. High *a*_CDOM_ values [~0.5 m^-1^ for *a*_CDOM_(443)] were observed in the central Arctic, which have been shown to have a high terrestrial signal [77], likely associated to transport of high-DOM Siberian Shelf waters [2] within the Transpolar Drift. That high-DOM signal can be traced even after significant removal during the transport of those waters to the central Arctic [20] and in the Fram Strait [22,23,30,74–76,78,79]. The lowest *a*_CDOM_ values [<0.2 m^-1^ for *a*_CDOM_(443)] were observed in the Beaufort Gyre and Amundsen and Nansen basins which is related to the influence of waters from the Norwegian and Barents Sea [45] that have a very low DOM content [78,80]. The low *a*_CDOM_(443) observed in the Beaufort Gyre corroborates the well-known DOM decrease towards the center of oligotrophic oceanic basins and gyres, where *a*_CDOM_(443) values tend to be close to zero [81,82]. Furthermore, *a*_CDOM_(443) differed significantly (*p*<0.001) between pelagic samples from the Central Arctic and Beaufort Gyre. Likewise, a recent study reported higher *a*_CDOM_(443) values in shelf waters of Eurasian basin in comparison to the Canadian basin [21]. Such a difference in the DOM background between the two basins is likely a reflection of the higher loads of DOM from Siberian Rivers [6,20]. Moreover, the differences between DOM from Eurasian and Canadian basins can be also detected in the intensity of visible DOM fluorescence, which can further distinguish the origins of fresh water exiting the Arctic Ocean [22].

We computed the total non-water coefficient absorption spectra [*a*_CDM_(λ)] as follows:

$$a_{tw}(\lambda )=a_{ph}(\lambda )+a_{NAP}(\lambda )+a_{CDOM}(\lambda ).$$ (13)

*a*_tw_(λ) was strongly correlated with *a*_CDOM_(λ) (*p*<0.0001) in the UV and VIS (violet-blue, mostly) wavelength ranges, suggesting CDOM as the major absorber component of the surface waters in that spectral regions through the entire sampling area (Fig 3C). Such dominance of CDOM is also clear when looking at the relative proportion of the non-water absorbers [*a*_ph_(443), *a*_NAP_(443) and *a*_CDOM_(443)] to *a*_tw_(443) (Fig 3B), which shows that all sampled stations are classified as CDOM-dominated [52]. The proportion of *a*_CDOM_(443) was high (Table 1), with it contributing to over 50% at all sampled stations, reaching a maximum contribution of 99% to *a*_tw_(443). Similar averaged values (0.85 ± 0.07) for the *a*_CDOM_(443) contribution to *a*_tw_(443) was observed in a recent study conducted in the Eurasian Basin [21]. Nearly as high contributions of CDOM were also reported for the Canadian Basin (Chukchi Sea: 0.74±0.14; Western Arctic: 0.76±0.11) in that same study [21]. Our study shows that CDOM is not only the major non-water absorber in the western Arctic and shelf seas of the eastern Arctic [21,35], it also strongly dominates the non-water absorption in the central Arctic. Dominance of CDOM to the total non-water absorption has been primarily reported to coastal environments, classified as “Case-2 waters” because CDOM (and also NAP) does not covary with Chl-*a* [83,84]. Oligotrophic pelagic systems (as the Central Arctic), on the other hand, are generally characterized as “Case-1 waters”, where Chl-*a* is thought to be the dominant absorber and covaries with CDOM [83,84]. That assumption, however, is not applicable to the pelagic Arctic Ocean, whose non-water absorption is clearly dominated by CDOM that, in turn, does not covary with Chl-*a* (*r*^2^ = 0.01). Such an absence of covariance between CDOM and Chl-*a* has been already reported for the Labrador Sea [85] and Western Arctic Ocean [35,86]. The correlation observed in this study (*r*^2^ = 0.01) was, nevertheless, the weakest observed for the entire Arctic Ocean, and could be related to the greater contribution and variability of CDOM to the total non-water absorption in our investigated waters. Finally, *a*_NAP_(443) contribution to *a*_tw_(443) was the lowest found for the Arctic waters, being likely negligible compared to *a*_CDOM_(443) contributions (Table 1).

**Table 1:** Relative absorption of non-water absorbers. Averaged contribution of the absorption coefficients for each of the non-water absorbers (at 443 nm) to *a*_tw_(443) in this and other studies carried out in different regions.

Study	Sampling area	Layer	$\frac{a_{ph}(443)}{a_{tw}(443)}$	$\frac{a_{NAP}(443)}{a_{tw}(443)}$	$\frac{a_{CDOM}(443)}{a_{tw}(443)}$	*n*
This study	Central & E Arctic	surface	0.12 ± 0.11	0.03 ± 0.02	0.85 ± 0.13	62
Matsuoka et al. (2014)	East Siberian and Laptev Seas	euphotic layer	0.08 ± 0.04	0.08 ± 0.02	0.85 ± 0.07	18
Matsuoka et al. (2014)	Chukchi Sea	euphotic layer	0.18 ± 0.12	0.08 ± 0.05	0.74 ± 0.14	179
Matsuoka et al. (2007)	Beaufort and Chukchi Seas	<90 m	0.16 ± 0.09	0.08 ± 0.03	0.76 ± 0.11	94
Kowalczuk et al. (2017)	North off Svalbard	<30 m	0.55 ± 0.2	0.04 ± 0.04	0.41 ± 0.22	19
Babin et al. (2003) [87]	Coastal Europe	surface	0.36 ± 0.14	0.22 ± 0.13	0.41 ± 0.14	317

### 3.3. Geographic clustering

**Fig 4:**
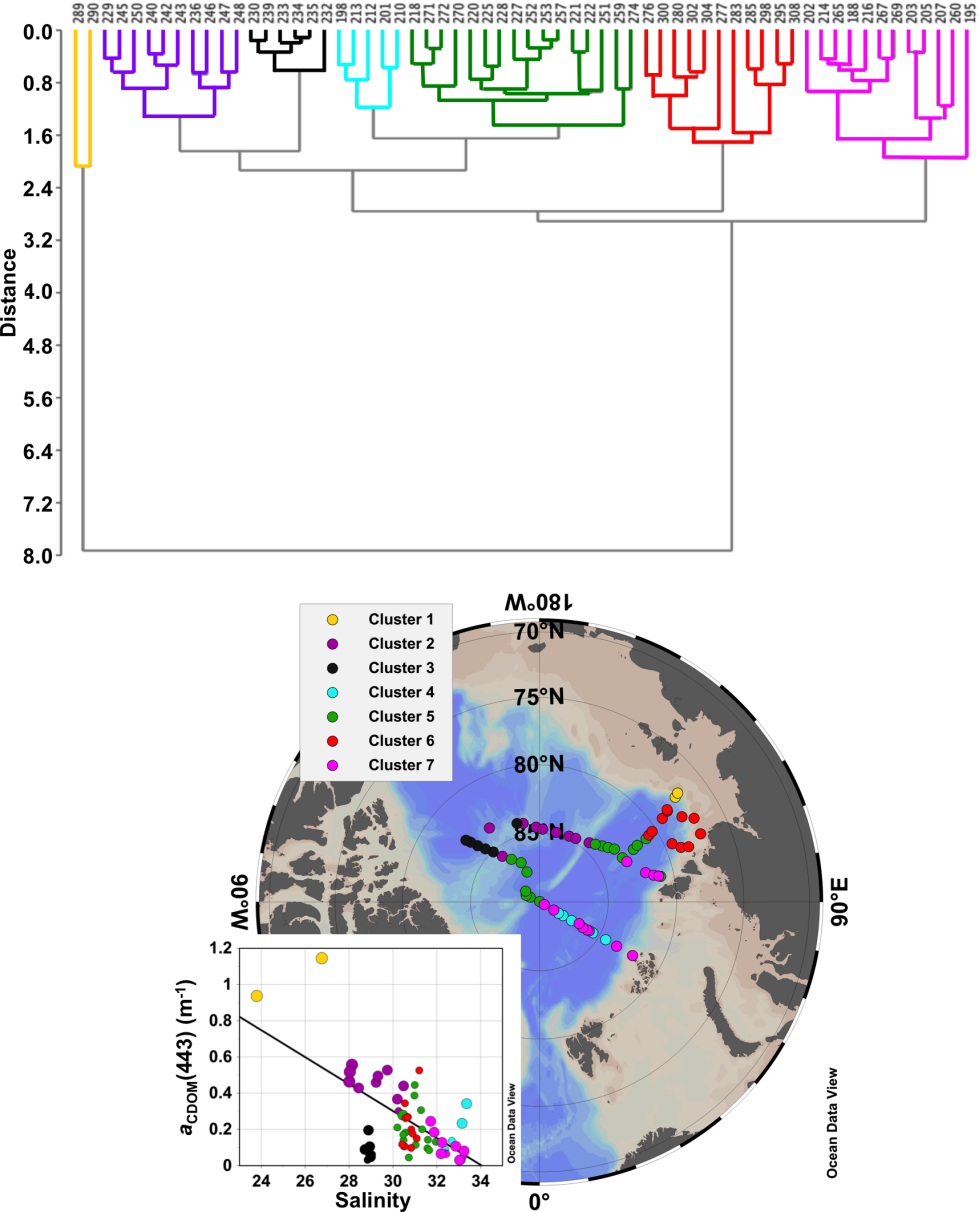
Clustering based on environmental matrix. (***top***) Dendogram (cophenetic correlation coefficient: *c* = 0.91) for sampling stations based on surface normalized values of an environmental matrix containing hydrographic and IOP parameters: temperature, salinity, *a*_CDOM_(443), *a*_NAP_(443) and *a*_ph_(443). (***bottom***) ODV map [44] showing the position of each station according to the classification based on the hierarchical clustering. Inset graph shows the correlation between *a*_CDOM_(443) and salinity colored with respect to the clusters; black line indicates the best fit (*p*<0.01).

Hierarchical cluster analysis was applied to the environmental matrix [temperature, salinity, *a*_CDOM_(443), *a*_NAP_(443) and *a*_ph_(443)] to classify the sampling sites according to coherent groups with respect to hydrography and non-water absorption. A total of seven major clusters were identified and those were used to divide the study area into five distinct geographic zones (Fig 4): Laptev Sea Shelf, Laptev Sea (pelagic), Central Arctic/Transpolar Drift, Beaufort Gyre and Nansen Basin. Those zones were easily discriminated based on the surface values of the environmental matrix. The average and standard deviation of the analyzed parameters for each cluster are presented in Table 2. In short, cluster 1 characterized the surface Laptev Sea shelf waters, influenced by the Lena River outflow, with high temperature, low salinity, moderate *a*_ph_(443) and the highest values of CDOM and NAP, in agreement with previous reports for that region [2,5,21,53,88]. Cluster 6 was composed by stations located in the pelagic and western domain of the Laptev Sea, with influence of shelf waters from the Kara Sea [71,72]. Those waters presented high temperatures, relatively low salinity and moderate values of *a*_CDOM_(443), *a*_ph_(443) and *a*_NAP_(443). Clusters 2 and 5 united the stations located in the Central Arctic, over the Transpolar Drift stream [45], where the Arctic shelf waters with relatively low salinity and high *a*_CDOM_(443) are transported along the Arctic Basin [20]; however cluster 5 seems to be a transitional zone, with less influence of Arctic shelf waters, exhibiting lower *a*_CDOM_(443) and higher *a*_ph_(443) compared to cluster 2. Cluster 3 grouped the stations located in the Beaufort Gyre. Those lower salinity waters [89] presented near freezing temperature and very low non-water absorption was observed, with *a*_CDOM_(443) and *a*_ph_(443) exhibiting the lowest values among the seven clusters. These results corroborate previous findings showing Canadian Basin water with low Chl-*a* and primary production [16,25], as well as lower DOM content [6,20–22], in comparison to the Eurasian Basin. Finally, the clusters 4 and 7 grouped the stations located in the Nansen and Amundsen basins, with influence of waters advected from the North Atlantic Ocean and Norwegian Sea. Those waters were characterized by the lowest temperatures, the highest salinity, low *a*_CDOM_(443) and *a*_NAP_(443), as reported for the waters of the Atlantic inflow to the Arctic in the Fram Strait [79]. On the other hand, *a*_ph_(443) (and Chl-*a*) values within that cluster were the highest, likely explained by the advection of nutrient rich Atlantic water [90] that stimulates phytoplankton growth. Clusters 4 and 7 differed from each other only regarding the *a*_ph_(443) (and Chl-*a*) values, with the highest values being observed in cluster 7. High *a*_ph_(443) (and Chl-*a*) observed in the Nansen and Amundsen basins can be attributed to the high transmittance of light in those waters primarily due to the development of melt ponds in the sea-ice [13], which increases primary production in those areas [24].

**Table 2:** Average of parameters for the geographic clusters based on the environmental matrix. Averaged values ± standard deviation of hydrographic/IOP parameters and geographic region for each of the clusters presented in Fig 4. Geographic regions acronyms: BG (Beaufort Gyre); EB (Eurasian Basin–Amundsen and Nansen basins); LS (Laptev Sea); LSS (Laptev Sea Shelf–Lena river influenced); TPD (Transpolar Drift).

Cluster	Temperature (°C)	Salinity	*a*_CDOM_(443) (m^-1^)	*a*_NAP_(443) (m^-1^)	*a*_ph_(443) (m^-1^)	*n*	Area
**1**	2.95 ± 0.15	25.2 ± 2.1	1.04 ± 0.15	0.04 ± 0.001	0.02 ± 0.001	2	LSS
**2**	-1.54 ± 0.05	29.2 ± 1.0	0.45 ± 0.08	0.003 ± 0.002	0.01 ±0.002	10	TPD
**3**	-1.47 ± 0.03	28.9 ± 0.1	0.09 ± 0.05	0.002 ± 0.0004	0.006 ± 0.001	6	BG
**4**	-1.71 ± 0.03	32.9 ± 0.4	0.16 ± 0.12	0.001 ± 0.001	0.01 ± 0.004	5	EB
**5**	-1.55 ± 0.23	31.0 ± 0.5	0.19 ± 0.11	0.005 ± 0.002	0.02 ± 0.004	16	TPD
**6**	0.99 ± 0.80	30.7 ± 0.2	0.23 ± 0.13	0.01 ± 0.003	0.02 ± 0.005	11	LS
**7**	-1.66 ± 0.14	32.4 ± 0.5	0.09 ± 0.06	0.005 ± 0.003	0.04 ± 0.01	12	EB

**Fig 5:**
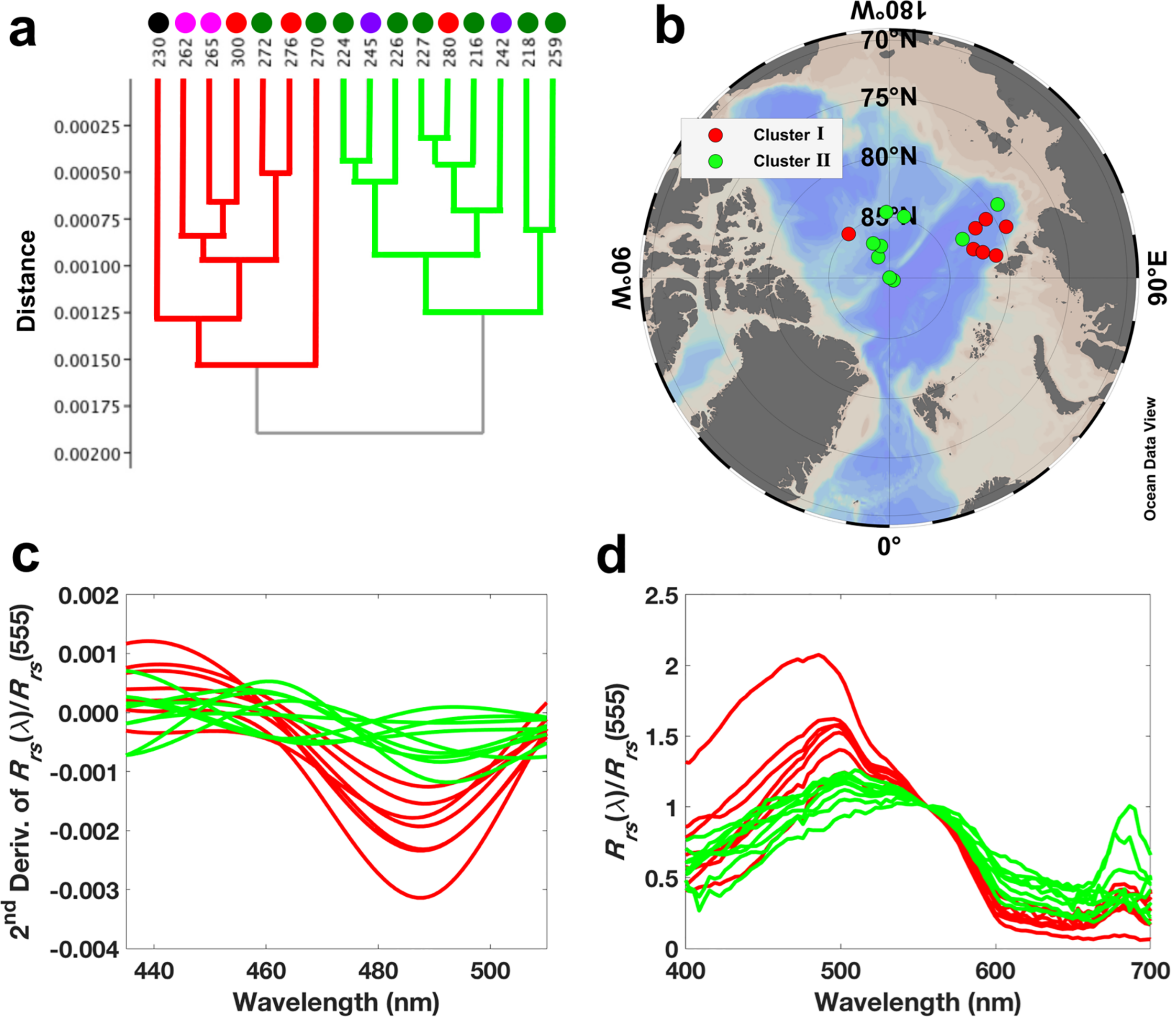
Hyperspectral AOP clustering. (**a**) Dendogram (cophenetic correlation coefficient: *c* = 0.87) for sampling stations based on hierarchical cluster analysis applied to the 2^nd^ derivative of *R*_rs_(λ)/*R*_rs_(555) (following Torrecilla et al., 2011). (**b**) ODV map [44] showing the position of each station according to the classification based on the hierarchical clustering. (**c**) 2^nd^ derivative of normalized hyperspectral remote sensing reflectance, *R*_rs_(λ)/*R*_rs_(555), with respect to the wavelength range of 435–510 nm (following Torrecilla et al. [65]). (**d**) Normalized hyperspectral remote sensing reflectance, *R*_rs_(λ)/*R*_rs_(555) in the visible wavelength range. Colored circles in (a) refer to the environmental clusters presented in Fig 4. Colors in (c) and (d) are in accordance with the clusters presented in (a) and (b).

To test whether hyperspectral remote sensing information is capable of detecting hydrographic and bio-optical variability we have also applied hierarchical cluster analysis to hyperspectral *R*_rs_ [in this case, the 2^nd^ derivative of *R*_rs_(λ)/*R*_rs_(555); see section 2.7]. Despite the low number of sampled stations (*n* = 16), the analysis yielded satisfactory results (cophenetic correlation coefficient: *c* = 0.87) and two main clusters were isolated (Fig 5). The partition based on hyperspectral data shows some similarities with the one provided by clustering the environmental matrix (see Fig 4). Cluster I comprised the *R*_rs_ spectra (i.e. stations) with lower *a*_CDOM_(443), located mainly in the Nansen and Amundsen basins and North Laptev Sea, under influence of waters from the North Atlantic, Norwegian Sea and also from Kara Sea. This cluster corresponds to the clusters 6 and 7 (and two stations of the transition cluster 5), with relatively low *a*_CDOM_(443) and influence of waters advected from the abovementioned regions. Additionally, the only station from the Beaufort Gyre, which also presented low *a*_CDOM_(443), was included in this same cluster I. Here we speculate that given the low number of stations performed, the multivariate analysis may not be able to solve such variability and grouped all the low CDOM spectra into one unique cluster. However, with an increased number of sampling stations, such variability would be easier to be detected in *R*_rs_ spectra. Cluster II isolated *R*_rs_ spectra from stations with high *a*_CDOM_(443) and lower *R*_rs_ (Fig 5 and Table 3), located in the central Arctic and close to the Laptev Sea shelf (Fig 5). Its corresponding environmental clusters are mainly the clusters 2 and 5, which were under influence of the shelf waters transported within the Transpolar Drift [45]. No stations of clusters 3 and 4 were sampled for hyperspectral remote sensing information.

**Table 3:** Hydrographic and IOP parameters for the geographic clusters based on hyperspectral AOP measurements. Averaged values ± standard deviation of geophysical parameters for each of the clusters presented in Fig 5.

Cluster	Temperature (°C)	Salinity	*a*_CDOM_(443) (m^-1^)	*a*_NAP_(443) (m^-1^)	*a*_ph_(443) (m^-1^)	*N*
**I**	-1.03 ± 0.86	31.1 ± 1.2	0.11 ± 0.03	0.01 ± 0.0002	0.02 ± 0.01	7
**II**	-1.30 ± 0.88	30.9 ± 0.9	0.31 ± 0.19	0.01 ± 0.003	0.02 ± 0.01	9

### 3.4. Arctic bio-optical provinces

**Fig 6:**
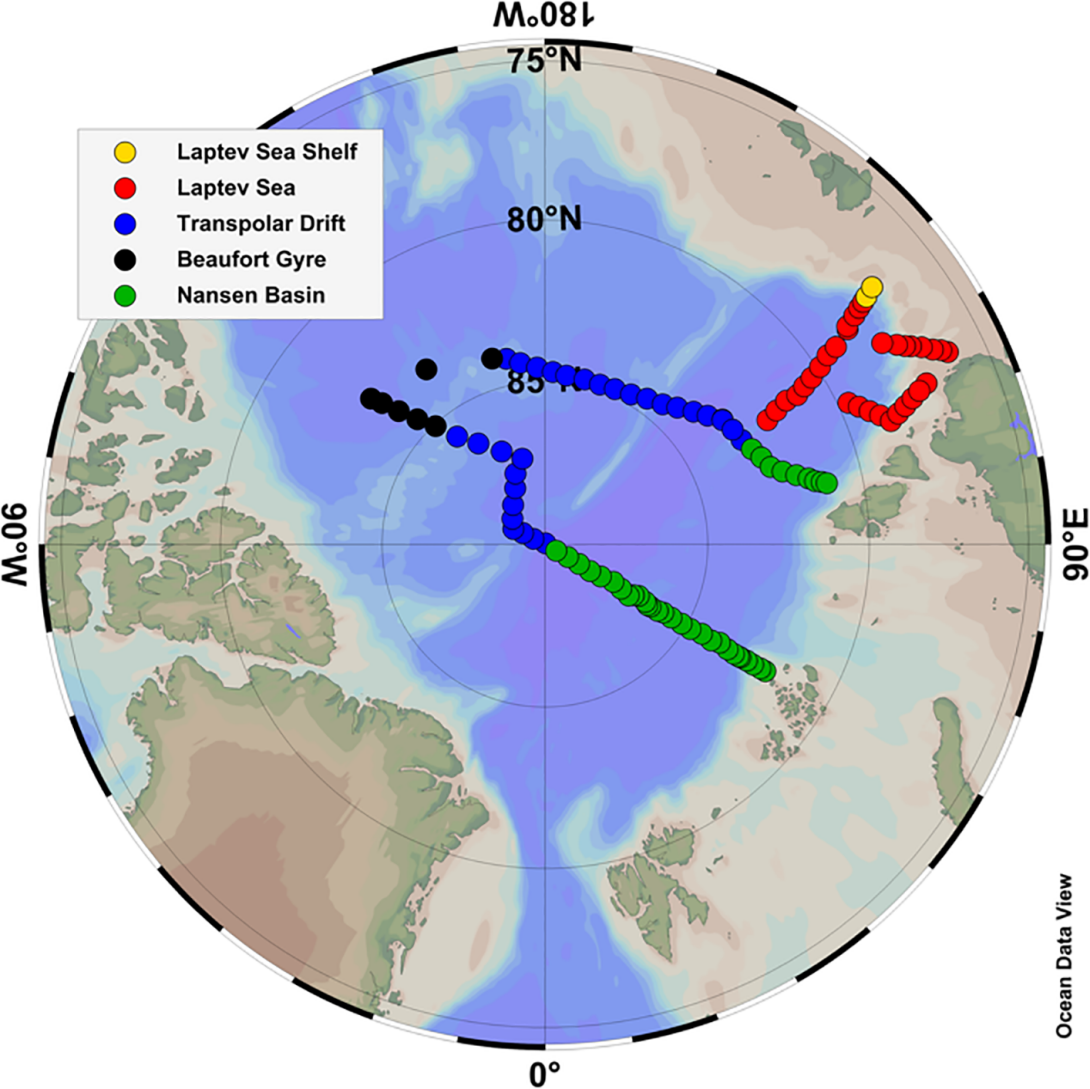
Arctic bio-optical provinces. Distribution of the five Arctic bio-optical provinces defined in this study based on HCA applied to surface hydrographical, IOP bulk and hyperspectral AOP data.

The results provided by hierarchical cluster analyses in this study (see Figs 4 and 5 and Tables 2 and 3) show that hydrographic data and non-water absorption, but also hyperspectral AOPs (e.g. *R*_rs_ spectra) are applicable tools for characterizing surface waters (geographic zones) with differing surface biogeochemical properties, even in waters where non-water absorption is strongly dominated by CDOM, such as the Arctic Ocean [21]. Similarly, a recent study applied hierarchical cluster analysis to the spectral particulate backscattering-to-absorption ratio in the western Arctic allowing the partitioning of optically-distinct clusters of particles assemblages, which, in turn, reflect difference in the characteristics of particle concentration, composition, and phytoplankton taxonomic composition and size [91]. Furthermore, given the coupling between hydrographic and bio-optical properties, one can further suggest those clusters as bio-optical units or provinces. Bio-optical provinces based on HCA applied to IOPs and AOPs have shown to be reliable describers of Longhurst provinces [92] in the Atlantic Ocean [54]. On the other hand, almost the entire Arctic Ocean is classified as a unique ecological province, the Boreal Polar Province (BPLR), within the Polar Biome [92]. That same author suggested that there might be spatial variability between shelf and pelagic ecosystems (as well as in the marginal ice zones) within the BPLR, however it is very difficult to sustain an adequate description of smaller units, given the difficulty to access the northern seas. Along with that, differences among the Arctic Seas have been already reported, for instance, with respect to export of biogenic matter [4], number of dinoflagellates species [27], protist diversity [28,29], and primary production [24,25,93]. Those studies, therefore, reinforce the existence of distinct biogeographic units in the Arctic Ocean and further implementation of a biogeographic characterization in the region is of great importance to improve the current understanding about the Arctic environment. The determination of such biogeographic zones would guide future strategies for Arctic monitoring and ecosystem modeling, leading to a more accurate understanding of the ecosystem functioning and biogeochemical stocks, as well as on the prediction of future scenarios with regards to climate change. Finally, to build on that, based on the results presented by our quasi-synoptic sampling through the central-eastern Arctic Ocean, we therefore propose an overall classification of the sampling sites into five major bio-optical provinces. Those sites were defined based on a combination of hydrographic characteristics and IOPs, but also considering the outcome of the hyperspectral AOP matrix clustering. They are classified as follows (Fig 6):

1. Laptev Sea Shelf: strongly influenced by the Lena River outflow, is primarily characterized by low salinity, high temperature and *a*_NAP_(443), moderate *a*_ph_(443), Chl-*a* and very high *a*_CDOM_(443);
2. Laptev Sea: low influence of Lena River outflow, however with contributions of waters advected from the Kara Sea; presents relatively low salinity, relatively high temperature and moderate levels of *a*_CDOM_(443), *a*_ph_(443), Chl-*a* and *a*_NAP_(443);
3. Central Arctic/Transpolar Drift: characterized by shelf waters transported within the Transpolar Drift, it has very low temperatures and relatively low salinity, *a*_ph_(443) and Chl-*a*; however with high *a*_CDOM_(443) and very low *a*_NAP_(443);
4. Beaufort Gyre: the waters with lower non-water absorption; they present low temperature and salinity, together with very low values of *a*_CDOM_(443), *a*_ph_(443), Chl-*a* and *a*_NAP_(443);
5. Eurasian/Nansen Basin: region influenced by waters advected from the Atlantic Ocean and Norwegian Sea, those waters present the highest salinity and near freezing temperature, with very low *a*_CDOM_(443) and *a*_NAP_(443), and the highest *a*_ph_(443) and Chl-*a* levels due to high transmittance through sea-ice.

### 3.5. Evaluation of ocean color algorithms

**Fig 7:**
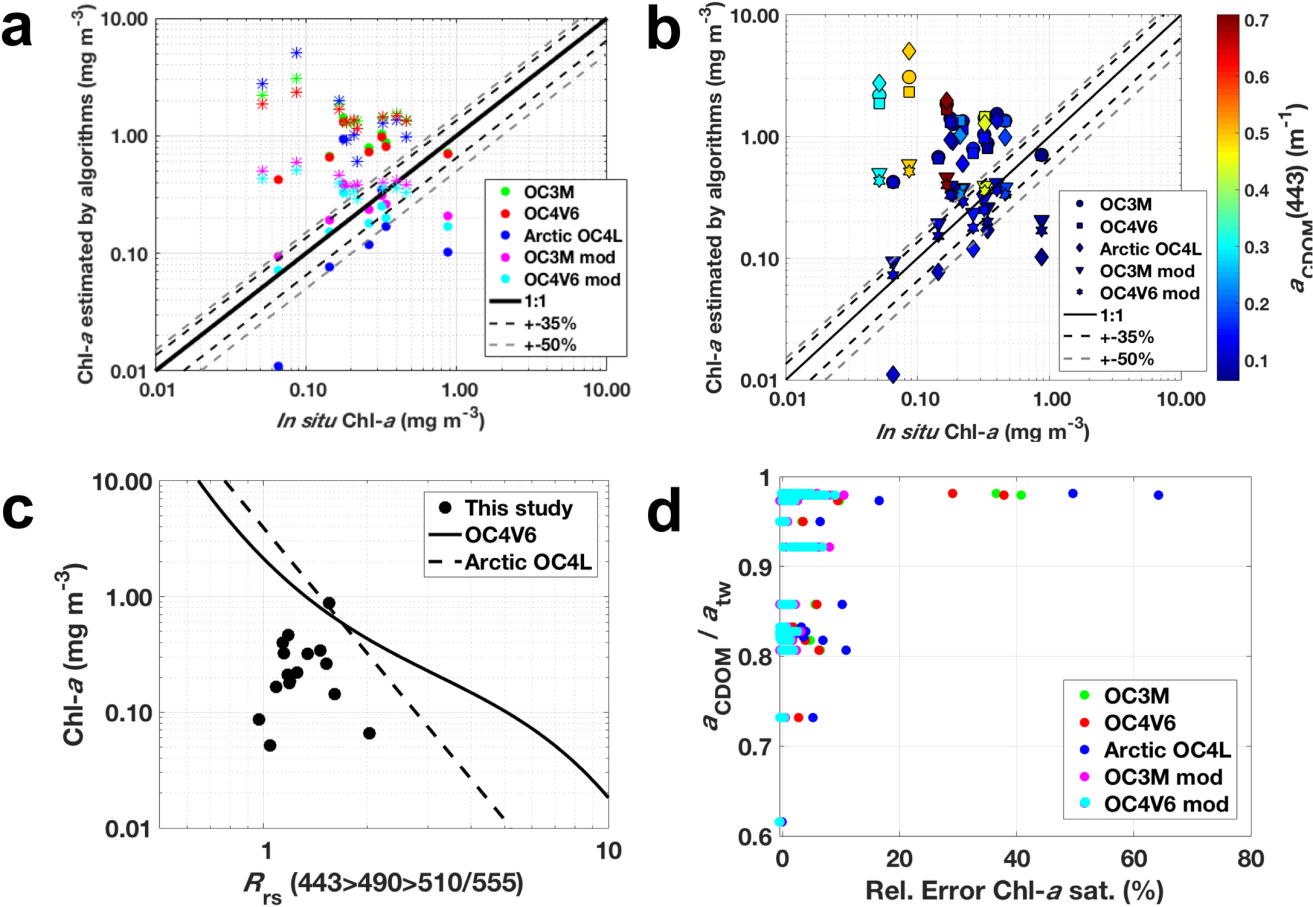
Evaluation of empirical ocean color algorithms frequently applied to the Arctic Ocean. **(a)** Chl-*a* estimated by empirical algorithms (mg m^-3^; indicated by different colors) versus *in situ* Chl-*a* (mg m^-3^). Stations belonging to the low *a*_CDOM_(443) cluster (Cluster 1) are presented as circles, whereas stars represent stations grouped in the high *a*_CDOM_(443) cluster (Cluster 2; Fig 5). **(b)** Chl-*a* estimated by empirical algorithms (mg m^-3^; indicated by different symbols) versus *in situ* Chl-*a* (mg m^-3^), with *a*_CDOM_(443) (m^-1^) as colorbar. **(c)** *In situ* Chl-*a* (mg m^-3^) versus maximum band ratio [MBR; *R*_rs_(443>490>510/555)]. **(d)** Chl-*a* estimated by empirical algorithms relative error (%) versus the ratio between *a*_CDOM_(443) and *a*_tw_(443).

Fig 7 shows the evaluation of the current global empirical ocean color algorithms OC3M and OC4V6 (and their regional adaptations for the western Arctic, OC3M-mod and OC4V6-mod) frequently applied to the Arctic Ocean, as well as the Arctic OC4L algorithm, which is designed to be applicable to high northern latitudes. When considering all sampled stations, the five empirical algorithms failed in retrieving Chl-*a* from *R*_rs_ bands, and a general inverse correlation with *in situ* Chl-*a* was observed (Fig 7 and Table 4). Furthermore, despite the relatively low RMSE observed for OC4V6-mod and OC3M-mod, all the band-ratio algorithms applied in this study appeared to attribute CDOM absorption to phytoplankton absorption (Fig 7D and Table 4). Such CDOM-biased retrievals from empirical Chl-*a* ocean color algorithms have already been reported for the western Arctic [33–35]. This is attributed to the fact that CDOM is the greatest absorber at 443 nm over the entire sampled region (see Fig 3 and Table 1). As pointed out by Chaves et al. [33], excess *a*_CDOM_(λ)–that is assumed to co-vary with Chl-*a*–produces lower maximum band ratios [*R*_rs_(443>490>510/555)], thus resulting in overestimation of Chl-*a* (see Fig 7C).

**Table 4:** Evaluation of empirical ocean color algorithms. Regression statistics (including the bias and the mean absolute error–MAE) for retrieved Chl-*a* from *in situ R*_rs_ compared to direct measurements of Chl-*a* using the correspondent algorithms versus in situ measured parameters. *r*^2^ and slope were calculated using log-transformed data for each of the correspondent parameters.

**Retrieved Chl-*a vs*. *in situ* Chl-*a***						
**Algorithm**	***N***	***r***^**2**^	**Slope**	**RMSE**	**MAE**	**BIAS**
**OC3M**	16	0.45	–0.14	0.62	1.06	1.06
**OC4V6**	16	0.38	–0.09	0.49	0.95	0.95
**Arctic OC4L**	16	0.29	–0.18	1.18	0.83	0.83
**OC3M-mod**	16	0.14	–0.01	0.13	0.08	0.08
**OC4V6-mod**	16	0.14	–0.01	0.12	0.03	0.03
**Retrieved Chl-*a vs*. *a***_**CDOM**_**(443)**						
**Algorithm**	***N***	***r***^**2**^	**Slope**	**RMSE**	**MAE**	**BIAS**
**OC3M**	15	0.83	0.49	0.49	1.06	1.06
**OC4V6**	15	0.82	0.44	0.36	0.96	0.96
**Arctic OC4L**	15	0.80	1.48	1.00	0.88	0.88
**OC3M-mod**	15	0.77	0.40	0.10	0.12	0.12
**OC4V6-mod**	15	0.78	0.44	0.09	0.07	0.07

A study in the western Arctic obtained good Chl-*a* retrievals applied to in situ *R*_rs_ measurements from CDOM-dominated waters (where Chl-*a* does not covary with CDOM), when turbid waters [*R*_rs_(676)>0.00042] were excluded [35]. This could be one of the reasons attributed to the poor performance of those algorithms in our study, given that all the sampling stations were classified as turbid. This is supported by the fact that the most overestimated Chl-*a* retrievals were especially related to the high CDOM cluster (see Fig 7). When looking only at the stations grouped in Cluster I, i.e. with lower *a*_CDOM_(443) (although still with relatively high turbidity), the retrievals were significantly improved (strong reduction in RMSE), primarily for OC4V6-mod and OC3M-mod (RMSE = 0.08 and 0.10, respectively), and positively correlated to *in situ* Chl-*a* (Table 5). Estimates were less variable (*r*^2^>0.62) compared to previously reported for the western Arctic [35]. Since there are no specific empirical algorithms to derive CDOM in the Arctic, we have applied to our data set the algorithm developed by Belanger et al. [39] for the Western Arctic to obtain the ratio of *a*_CDOM_ to *a*_dg_ in optically complex waters. However, we also did not obtain robust results using this method.

**Table 5:** Comparison of Chl-*a* retrieved from empirical ocean color algorithms versus direct measurements of Chl-*a*, for low *a*_CDOM_(443) sites. Same as Table IV but for the low *a*_CDOM_(443) stations.

Retrieved Chl-*a vs*. *in situ* Chl-*a*						
Algorithm	*N*	*r*^2^	Slope	RMSE	MAE	BIAS
**OC3M**	7	0.62	0.18	0.34	0.83	0.83
**OC4V6**	7	0.62	0.17	0.30	0.77	0.77
**Arctic OC4L**	7	0.66	0.75	0.35	0.25	0.25
**OC3M-mod**	7	0.70	0.28	0.10	0.01	–0.01
**OC4V6-mod**	7	0.70	0.29	0.08	0.08	–0.08

**Fig 8:**
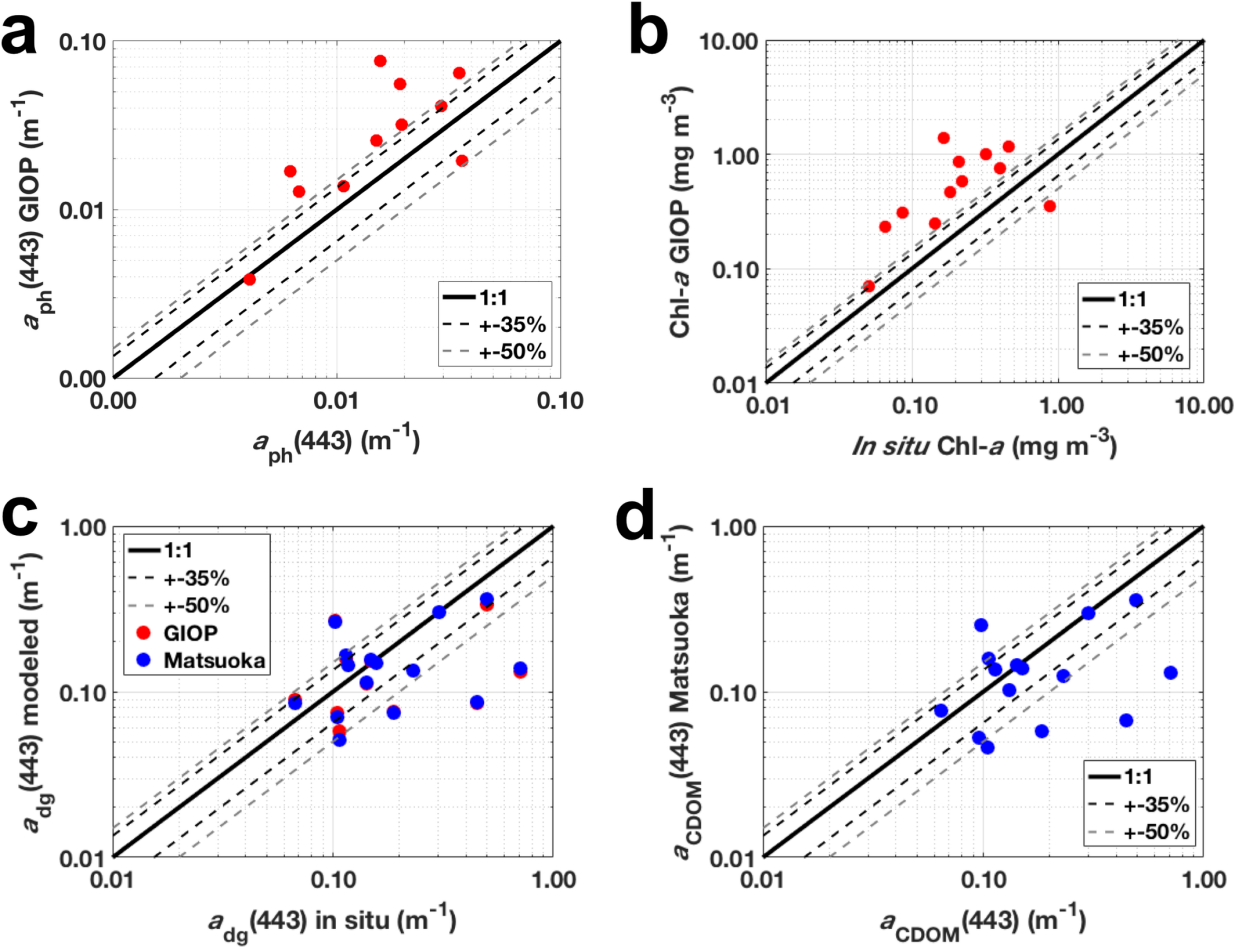
Evaluation of semi-analytical algorithms. Modeled geophysical parameters calculated from *in situ R*_rs_ versus *in situ* measured parameters: *a*_ph_(443) **(a)**; Chl-*a* **(b)**; *a*_dg_(443) (**c**) and *a*_CDOM_(443) **(d)**. Red points refer to the GIOP [60,61] retrievals, whereas blue points to the retrievals from the GSM model adapted to the Arctic [19].

Besides, the SAA GIOP was applied to retrieve Chl-*a*, as well as *a*_ph_(443) and *a*_dg_(443). This provided robust estimates (Fig 8) for the entire sampling area (Fig 8 and Table 6). Such an improvement probably is caused by that GIOP, like other SAAs, does not assume Chl-*a* and CDOM absorption as covariant. Even better estimates from GIOP were obtained for *a*_ph_(443) (Fig 8 and Table 6). This is probably due to the fact that GIOP uses the spectral shape of Chl-*a*-specific absorption coefficient from Bricaud et al. [68] as basis vector. As pointed out before (Fig 3), our dataset exhibited similar spectral shape for *a*_ph_(λ) and correlations between Chl-*a* and *a*_ph_(443) as observed in that study. Moreover, the performance of GIOP to retrieve *a*_ph_(443) in our study (Table 6) was much better than recently observed in the western Arctic (*r*^2^ = 0.85; Slope = 1.18; RMSE = 0.20) [33]. With regards to Chl-*a*, that same study reported fairly similar results (*r*^2^ = 0.72; Slope = 0.73; overestimation of Chl-*a*), however with lower errors (RMSE = 0.24) in comparison to our results (RMSE = 0.40, see Table 6).

**Table 6:** Evaluation of the semi-analytical ocean color algorithms. Regression statistics for modeled geophysical parameters calculated used *in situ R*_rs_ versus in situ measured parameters. *r*^2^ and Slope were calculated using log-transformed data for each of the correspondent parameters.

	*N*	*r*^2^	Slope	RMSE	MAE	BIAS
**GIOP**						
***a***_**ph**_**(443)**	11	0.86	0.89	0.02	0.02	0.02
**Chl-*a***	12	0.79	0.64	0.40	0.35	0.35
***a***_**dg**_**(443)**	15	0.56	0.25	0.08	0.08	–0.08
**GSM-Mat**						
***a***_**dg**_**(443)**	15	0.59	0.29	0.09	0.07	–0.07
***a***_**CDOM**_**(443)**	15	0.57	0.28	0.09	0.08	–0.08

Finally, *a*_dg_(443) was also retrieved in this study using GIOP and GSM-Matsuoka. Here we assume that *a*_dg_(443) is a direct estimate from of *a*_CDOM_(443), given the least contribution of *a*_NAP_(443) to total non-water absorption (generally <1%) in comparison to *a*_CDOM_(443) (Fig 3). In general, GIOP and GSM-Matsuoka retrievals were very similar and the retrieved *a*_dg_(443) compared very well to direct measurements of *a*_dg_(443). Most of the data points located are within the 50% error intervals (Fig 8 and Table 6). Although with similar error (RMSE = 0.08), GIOP seems to provide more robust correlation (slope = 1.05) and less variable (*r*^2^ = 0.91) *a*_dg_(443) estimates for the western Arctic, as observed for *a*_ph_(443) [33]. As being highly correlated to *a*_dg_(443), *a*_CDOM_(443) retrieved with GSM-Matsuoka provided very similar statistics related to *a*_dg_(443) using that same model, thus resembling the observed with our *in situ* sampling (see Table 1). Compared to our study, better performance for retrieving *a*_CDOM_(443) using GSM-Matsuoka (*r*^2^ = 0.87; Slope = 0.97; RMSE = 0.07), was reported for the western Arctic using a much larger dataset [21]. Although the *a*_CDOM_(443) GSM-Matsuoka retrievals were not as good in our study, results show that SAAs in general have a high potential for obtaining reliable Chl-*a* estimates than empirical algorithms in high CDOM containing waters, besides the advantages of also providing other reliable retrievals such as *a*_dg_(λ), *a*_ph_(λ) and *a*_CDOM_(λ). Thus, products from SAAs are more suitable for application to biogeochemical studies in the Arctic Ocean, although improvement of the current algorithms is still requested, given the persistence of embedded errors to those retrievals, as demonstrated in this study.

## 4. Summary and outlook

Based on a quasi-synoptic sampling strategy over the surface Central-Eastern Arctic Ocean we reiterate the dominance of CDOM related to total non-water absorption through the entire region under study. As CDOM and DOC are strongly correlated in the Arctic Ocean [6,20,53], one can assume CDOM as a very reliable proxy for retrieving carbon concentrations in that basin. This can provide additional insight into the Arctic biogeochemical cycles. Our results show that *a*_CDOM_(443) and *a*_ph_(443), together with temperature and salinity, are useful parameters for distinguishing hydrographic regimes within the Central Arctic. Despite the reduced number of sampling sites, hyperspectral AOPs retrieved from under water radiometric measurements were able to reproduce the major bio-optical features at the surface by differentiating between sites with low and high CDOM. As demonstrated for the Eastern Atlantic [54], bio-optical provinces efficiently reflected the ecosystem variability/biogeography proposed by Longhurst [92] and thus are a valuable tool for biogeochemical modeling. However, currently, practically the entire Arctic Ocean is still classified as a unique ecosystem unit, despite the reports of clear geographic patterns in different aspects [4,24,29,93,94]. Moreover, to our understanding, no study has proposed such a sub-division of the Boreal Polar Province (BPLR) into bio-optical units. Based on our findings we, therefore, propose a geographical characterization of the sampling regions into bio-optical provinces, which reflect hydrographic characteristics of the region with regard to the non-water absorption: Laptev Sea Shelf, Laptev Sea, Central Arctic/Transpolar Drift, Beaufort Gyre, and Eurasian/Nansen Basin. Moreover, it becomes clear that the characterization of provinces, in particular, in the highly seasonal variable Arctic Ocean, cannot hold true for every season and every year. Thus, although here we present a first, simple bio-optical classification, we recall that such variability has been observed along the Arctic and integrative biogeochemical studies would benefit from the advances in Arctic Ecosystem monitoring and management by improving the delimitation of such geographic units. Future perspectives using automated platforms (e.g., floats, ITP, gliders) with bio-optical (e.g., Chl-*a* and DOM fluorescence, and hyperspectral radiometry) and salinity sensors will allow to monitor the spatial and temporal variability within those biogeographic provinces.

The evaluation of empirical ocean color algorithms (including the regionally tuned ones) applied to our *in situ R*_rs_(λ) measurements showed that those algorithms are inappropriate to estimate Chl-*a* in the Central-Eastern Arctic Ocean, exhibiting an overall inverse correlation with *in situ* Chl-*a* and a positive correlation with *a*_CDOM_(443). This reinforces the existence of a persuasive positive bias by CDOM absorption on empirical Chl-*a* estimates for the Arctic Ocean. The semi-analytical ocean color algorithm GIOP, on the other hand, retrieved reliable and less variable Chl-*a* estimates related to the empirical algorithms, as well as very good estimates for *a*_ph_(443) and considerably well estimates of *a*_dg_(443), as also reported to the western Arctic [33]. Fairly similar retrievals were obtained within the GSM model with the modifications for the Arctic Ocean [19] for *a*_dg_(443). The better performance by SAAs is mainly attributed to the fact that these algorithms do not consider Chl-*a* and CDOM as covariant.

Finally, with the ongoing pressure of climate change over the Arctic environment, a better understanding on the dynamics of carbon stocks has been sought. Ocean color remote sensing appears to be a key tool on improving both the spatial and temporal monitoring of these stocks. However, accurate ocean color retrievals are required to get to a real estimate of stocks and processes involving organic and inorganic carbon in the Arctic Ocean. Thus, we recall that additional spectral bands would improve the performance of ocean color algorithms, as demonstrated for the GIOP in the western Arctic [33]. In addition, the coverage of ocean color remote sensing data in the Polar Regions needs to be increased (for our cruise we obtained no match-ups with satellite data) by investing in developing efficient atmospheric correction for adjacency effects and low illumination conditions.
